# The HGF/MET Axis in Advanced Prostate Cancer: From Context-Dependent Biology to Biomarker-Driven Therapeutic Strategies

**DOI:** 10.3390/cancers18091463

**Published:** 2026-05-02

**Authors:** Filippos Koinis, Maria Smaragdi Vlachou, Georgios Nintos, Georgios Christodoulopoulos, Emmanouil Panagiotidis, Ioannis Eleftheropoulos, Galatea Kallergi, Michail Samarinas, Athanasios Kotsakis

**Affiliations:** 1 Laboratory of Oncology, Faculty of Medicine, School of Health Sciences, University of Thessaly, GR-41110 Larissa, Greece; thankotsakis@uth.gr; 2 Department of Medical Oncology, University General Hospital of Larissa, 41110 Larissa, Greece; smarovlachou96@gmail.com (M.S.V.); georgenintos@gmail.com (G.N.); gechrist@uth.gr (G.C.); 3 Department of Nuclear Medicine, Faculty of Medicine, School of Health Sciences, University of Thessaly, 41110 Larissa, Greece; manospanagiotidis@uth.gr; 4 Department of Urology, General Hospital Asklipieion of Voula, 16673 Voula, Greece; i.eleftheropoulos@gmail.com; 5 Laboratory of Biochemistry and Metastatic Signaling, Department of Biology, University of Patras, 26504 Patras, Greece; gkallergi@upatras.gr; 6 2nd Department of Urology, Medical School, Aristotle University of Thessaloniki, 54124 Thessaloniki, Greece; msamaria@auth.gr

**Keywords:** prostate cancer, MET pathway, precision medicine, targeted treatment, cabozantinib, AR-indifferent, aggressive prostate cancer, biomarkers

## Abstract

**Background/Objectives:** Advanced prostate cancer (PCa) evolves through adaptive mechanisms that sustain tumor growth despite the suppression of androgen receptor (AR) signaling. Accumulating evidence identifies activation of the hepatocyte growth factor (HGF)/MET pathway as a potential driver of PCa progression in advanced disease states characterized by AR-independence and therapeutic resistance. We review the biological and clinical evidence supporting MET as a context-dependent therapeutic target and discuss its implications for patient selection and combination strategies. **Methods:** A comprehensive narrative review of preclinical, translational, and clinical studies evaluating MET-directed therapies for PCa was performed. **Results:** Aberrant activation of the HGF–MET axis is frequently driven by autonomous paracrine and autocrine loops that sustain pathway activation during disease progression. MET overexpression is associated with adverse pathological features, increased tumor aggressiveness, bone metastasis, lineage plasticity, and resistance to AR-targeted treatments. Preclinical studies have demonstrated that AR suppression, tumor hypoxia and tumor–microenvironment interactions promote MET upregulation, supporting AR-independent growth and epithelial-to-mesenchymal transition. Clinical trials of MET inhibitors have shown modest activity as monotherapies, with the most consistent biological effects observed in bone-dominant disease. Recent studies indicate greater therapeutic potential when MET inhibition is incorporated into rational combination strategies targeting complementary molecular pathways. Emerging data further indicate that MET activation characterizes a biologically aggressive, AR-low or neuroendocrine-like disease state. These findings support a transition from empiric use of MET inhibitors toward precision, context-driven therapeutic development. **Conclusions:** MET is not a universal therapeutic target but defines a clinically relevant subset of aggressive, AR-indifferent PCa. Future development should focus on biomarker-guided patient selection and rational combination strategies. Integration of molecular profiling, imaging, and liquid biopsy approaches will be essential to identify patients most likely to benefit from MET-directed interventions.

## Simple Summary

Despite therapeutic advances, metastatic prostate cancer remains a leading cause of cancer-related death in men. As tumors escape hormonal control, they rewire their signaling dependencies activating alternative survival pathways that sustain growth independently of androgen receptor activity. Among these, the HGF/MET signaling axis has emerged as a biologically compelling but clinical elusive target, enriched in aggressive, treatment-resistant disease states characterized by lineage plasticity and neuroendocrine differentiation. Herein, we critically examine the preclinical and clinical evidence supporting MET as a context-dependent therapeutic vulnerability, interrogate the reasons for the modest efficacy of MET-directed therapies in unselected patient populations, and propose a precision framework centered on biomarker-driven patient selection and rational combination strategies. We argue that realizing the therapeutic potential of this pathway will require moving beyond empiric broad application toward molecularly informed trial design that align specific interventions with defined disease contexts.

## 1. Introduction

Prostate cancer (PCa) is the second most frequently diagnosed cancer and the fifth leading cause of cancer-related death among men globally [1]. Despite the significant improvement in overall survival (OS) observed over recent decades, patients with metastatic PCa continue to experience low cure rates. Current life-prolonging treatment strategies rely primarily on targeting androgen receptor (AR) signaling and administering chemotherapy and bone-targeted radiopharmaceuticals [2]. Despite extensive efforts and innovative trial designs, the range of therapeutically exploitable targets in PCa has remained largely unchanged, and existing treatment modalities appear to have reached the limits of their effectiveness [3]. A clinically meaningful improvement in survival will likely necessitate the development of novel treatment strategies with different biological rationales. The encouraging advances in clinical outcomes for patients with metastatic castration-resistant PCa (mCRPC) achieved through prostate-specific membrane antigen (PSMA)-targeted radionuclide therapies, poly(ADP-ribose) polymerase (PARP) inhibitors in tumors with DNA damage repair alterations, and immune checkpoint inhibitors in specific subgroups further support this observation [4].

Translational and clinical research has established a role for the hepatocyte growth factor (HGF)/MET signaling pathway in PCa progression across both the epithelial-tumor and the microenvironmental compartments. MET expression is detected in primary prostate tumors and is further enriched in bone metastases and metastatic sites from patients with mCRPC. MET functions as a central signaling node within a network of interlinked oncogenic pathways governing PCa cell transformation, proliferation, invasion, migration, and metastasis. In vivo studies have also implicated MET in the development of treatment resistance in PCa, particularly in advanced mCRPC. Emerging evidence indicates that MET is not a universal oncogenic driver but a context-dependent vulnerability, enriched in aggressive disease states characterized by microenvironmental signaling, lineage plasticity, and relative AR independence. Clinically, these biological features often correspond to specific disease phenotypes, including rapid progression following AR pathway inhibition, discordance between tumor burden and PSA levels, bone-dominant metastatic disease, and treatment-emergent neuroendocrine (NE) or AR-low disease states. Collectively, in light of its prognostic significance, MET has emerged as an attractive therapeutic target. However, most clinical trials of MET inhibitors have been conducted in molecularly unselected populations, which may partly explain their limited efficacy and underscores the need for biomarker-driven patient selection in future mCRPC studies. Although several reviews have addressed individual aspects of MET biology in PCa, a clinically oriented synthesis integrating disease context, clinical trial outcomes, resistance mechanisms, and implications for patient selection is currently lacking. Herein, we aim to bridge this gap by reviewing the biological and clinical evidence linking MET to PCa progression, critically appraising the completed clinical trials, and proposing a context-dependent framework for future therapeutic development grounded in biomarker-driven patient selection and rational combination strategies. Although the HGF/MET axis is considered in its entirety, greater emphasis is placed on MET given its role as the principal therapeutically actionable component of the pathway.

## 2. MET/HGF Pathway Structure and Canonical Function

The MET proto-oncogene, located on chromosome 7q21-31, encodes a 170 kDa precursor polypeptide that undergoes proteolytic cleavage to generate two distinct subunits [5,6]: an extracellular α-subunit and a heavier, transmembrane β-subunit, which remain covalently linked through a disulfide bridge. This cleavage does not fully segregate these subunits, as the β-subunit retains an extracellular segment responsible for ligand binding in addition to a transmembrane and an intracellular domain. The intracellular domain harbors key regulatory elements, including a juxtamembrane region and a catalytic tyrosine kinase domain, which collectively enable precise control over receptor activation and downstream signaling [7,8,9]. Its cognate ligand, HGF, is secreted by mesenchymal cells as an inactive molecule and is converted to its biologically active form via proteolytic cleavage by serum activators and type II cellular serine proteases. Thus, mature HGF is a 130 kDa heterodimer composed of two distinct polypeptide chains [10].

MET is normally expressed in the epithelial cells of multiple organs, including the liver, pancreas, prostate, and kidney, as well as in muscle and bone marrow. HGF functions primarily as a cytokine that stimulates cell proliferation and promotes cell survival. Indeed, in vitro and in vivo animal studies indicate a potentially protective role for the signaling axis in normal liver tissue by facilitating regeneration in conditions such as liver fibrosis and cirrhosis through the modulation of inflammatory responses [11,12,13]. Under canonical conditions, MET is activated by the binding of endogenous HGF, which leads to receptor dimerization. Once dimerized, the MET receptors cross-phosphorylate each other at specific tyrosine residues (e.g., Y1234, Y1235, Y1349, and Y1356), triggering downstream activation of the tyrosine kinase cytoplasmic domain and creating docking sites for other signaling proteins [14].

**Figure 1:**
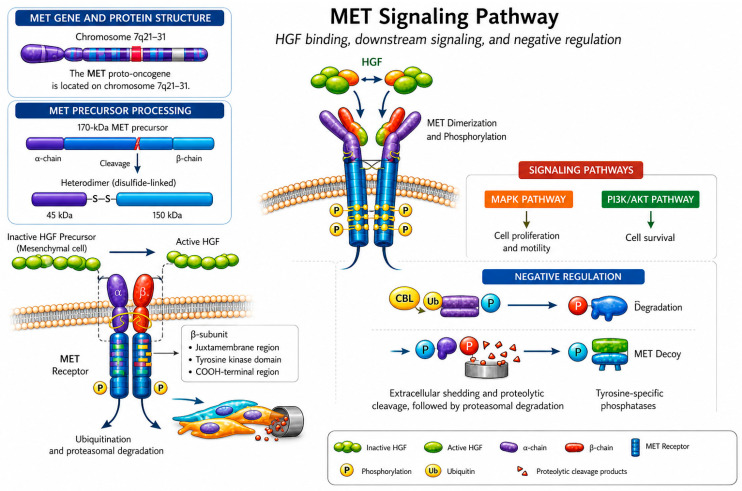
**Overview of MET–HGF signaling and regulatory mechanisms.** Schematic representation of MET receptor activation by hepatocyte growth factor (HGF) and the principal downstream signaling pathways. Binding of active HGF induces MET dimerization and receptor autophosphorylation, leading to activation of the MAPK and PI3K–AKT pathways, which regulate cell proliferation, motility, and survival. MET signaling also engages SRC–FAK complexes to promote cell migration and invasion. Negative regulatory mechanisms, including receptor ubiquitination, proteasomal degradation, dephosphorylation by tyrosine-specific phosphatases, and extracellular shedding of MET, limit signaling intensity and duration.

MET activity is further regulated through interactions with intracellular mediators, such as phosphoinositide 3-kinase (PI3K), phospholipase Cγ1 (PLCγ1), growth factor receptor-bound protein 2 (GRB2), GRB2-associated binding protein 1 (GAB1) and signal transducer and activator of transcription 3 (STAT3) [15,16]. More recent studies have identified additional biochemical contributors to this modulatory process, including Src homology-2-containing (SHC), v-crk sarcoma virus CT10 oncogene homolog (CRK), CRK-like (CRKL), v-src sarcoma viral oncogene homolog (SRC) and Src homology domain-containing 5′ inositol phosphatase (SHIP-2). Dysregulation of these factors has been reported to initiate molecular cascades that propagate cancer-related traits, including uncontrolled cell growth, inhibition of apoptosis and eventually metastasis, as the disease progresses [17,18,19]. Mechanistically, MET is considered a crucial signaling hub that orchestrates the activation of a broad spectrum of downstream signaling pathways that govern diverse cellular processes. For example, in vitro studies have reported that stimulation of the mitogen-activated protein kinase (MAPK) pathway activates transcription factors regulating genes that promote cell proliferation and motility [20,21]. Cellular survival is supported through the recruitment and activation of the p85 subunit of PI3K by MET, underscoring the essential role of MET in sustaining cell viability [22]. MET-mediated signaling also facilitates cell migration, specifically through interactions with SRC and focal adhesion kinase (FAK) [23]. Consistent with these findings, Zhu et al. demonstrated that MET activation enhances cell motility by disrupting cadherin-dependent cell–cell junctions [24]. In parallel, MET activation by HGF is tightly constrained by multiple negative regulatory mechanisms that restrict or even terminate uncontrolled pathway activity [25]. Both Casitas B lineage lymphoma (CBL)-mediated ubiquitination and subsequent degradation of the MET receptor, as well as MET dephosphorylation through the action of tyrosine-specific phosphatases, represent two major attenuation pathways [26,27]. Moreover, extracellular shedding and proteolysis of MET generate a soluble receptor fragment that is rapidly degraded by the proteasome; intriguingly, this fragment can also bind HGF, forming a decoy complex that antagonizes full-length receptor signaling [28] (Figure 1). Thus, under canonical conditions, the balanced interplay between positive and negative regulators preserves cellular homeostasis; however, disruption of this equilibrium can lead to aberrant signaling that may promote malignant transformation.

## 3. MET Pathway in Cancer

The HGF/MET complex is a tightly regulated system that plays a critical role in the maintenance of tissue homeostasis. However, it is susceptible to various processes that lead to constitutive pathway activation and thus disruption of normal cell behavior. These processes arise through both cell-autonomous and non-cell-autonomous mechanisms [29].

### 3.1. Tumor-Intrinsic Genetic Mechanisms of MET Activation

The oncogenic role of MET was first demonstrated by Cooper et al. in human osteosarcoma, where a chromosomal rearrangement resulted in fusion of the MET tyrosine kinase domain, leading to sustained receptor activation [30]. This translocation, also validated in animal models, results in the formation of an upstream promoter region that has been implicated in the development of human gastric carcinoma [31,32]. Further investigations have demonstrated that MET amplification and overactivation occur across several tumor types. Gastrointestinal carcinomas—including gastric, esophageal and colon cancers, particularly those with liver metastasis—are among the most extensively studied, although abnormal MET expression has also been reported in medulloblastomas [33,34]. More recently, MET dysregulation has gained attention in lung cancer, where protein overexpression, gene amplification and exon 14-skipping alterations have been identified as key oncogenic drivers [35]. MET activation in this context may represent a marker of tumor progression and resistance to conventional treatment, linked to both primary and acquired resistance to epidermal growth factor receptor (EGFR)-targeted therapies through bypass signaling mechanisms [36].

Beyond sporadic malignancies, MET also plays a critical role in hereditary cancer syndromes. Early evidence from the late 1990s demonstrated that activating mutations of the MET proto-oncogene are crucial drivers of hereditary papillary renal cell carcinoma. A novel germline MET mutation (V1110I) in the ATP binding site of the kinase domain was identified in affected family members and conferred transformative activity in vitro, highlighting its oncogenic potential [37]. Additional missense mutations in the tyrosine kinase domain often inherited as heterozygous alterations on chromosome 7 have also been described; however, tumorigenesis generally requires increased gene dosage through duplication or amplification of the mutant allele, which is consistent with a “dosage-dependent” mechanism. Although less frequent, oncogenic MET alterations are not confined to the tyrosine kinase domain but may also affect the juxtamembrane domain or the HGF binding site [38,39]. These observations underscore the diverse structural mechanisms through which MET genetic alterations can contribute to human oncogenesis.

### 3.2. Microenvironment-Dependent Mechanisms of MET Upregulation

MET signaling can be aberrantly upregulated through microenvironment-dependent mechanisms. The interplay between tumor hypoxia and the MET pathway has been extensively investigated. Hypoxic conditions in the tumor microenvironment (TME) trigger the transcription of hypoxia inducible factor 1-alpha (HIF-1α), which in turn activates the MET promoter [40]. This mechanism has been demonstrated across multiple tumor-derived cell lines, including lung, ovarian, cervical, and hepatocellular carcinoma, as well as osteosarcoma. This process can result in sustained pathway activation in the absence of gene amplification by enhancing the responsiveness of the MET receptor to its ligand, HGF [41]. In the bone microenvironment, where hypoxia is prevalent, this mechanism may contribute substantially to MET-driven bone tropism and metastatic progression.

MET overactivation can also be induced through HGF (ligand) and not receptor protein overexpression per se. Integrated preclinical and clinical data have revealed the presence of autocrine and paracrine loops that contribute to dysregulated HGF expression within the TME. This dysregulated signaling circuit has been associated with tumor aggressiveness, epithelial-to-mesenchymal transition (EMT) and poor clinical outcomes [42,43].

## 4. MET Pathway in PCa

### 4.1. MET Expression Across Disease Stages

The HGF/MET axis is critically involved in the oncogenic progression and metastatic dissemination of PCa. In benign prostate tissue, MET protein expression is low and is confined mainly to basal cells; however, it is progressively upregulated during malignant transformation, becoming detectable in high-grade prostatic intraepithelial neoplasia (PIN), present in most primary adenocarcinomas, and nearly universal in lymph node and bone metastases [44]. MET expression is consistently correlated with a high Gleason score, advanced disease stage, hormone-resistant disease state, and poor clinical outcome [45]. Increasing evidence indicates that MET overexpression and enhanced activity, both implicated in disease progression and emergence of CRPC, predominantly arise through copy-number independent mechanisms, including transcriptional activation, post-transcriptional regulation, and/or increased ligand availability within the TME [46].

### 4.2. AR–MET Crosstalk and Regulation of Expression

Preclinical studies suggest that AR negatively regulates MET expression by repressing MET transcription via the inhibition of Sp1-driven promoter activity, resulting in low MET levels in AR-active and androgen-dependent cells. Conversely, loss of AR signaling relieves this suppression and leads to MET overexpression, contributing to a low AR/PSA and high MET phenotype in hormone-refractory lesions [47]. Thus, androgen deprivation therapy (ADT) and AR pathway inhibitors (ARPIs) may induce MET expression and select for MET-high, AR-indifferent and hormone-resistant clones [48].

### 4.3. MicroRNA–Mediated Regulation

MicroRNAs (miRNAs) have emerged as additional post-transcriptional regulators of MET expression in PCa, and the downregulation of several tumor-suppressive miRNAs has been linked to elevated MET. Specifically, MET has been identified as a direct target of miR-493-5p, miR-200b, miR-205, and miR-34; downregulation of these miRNAs in AR-negative cell lines (PC3, DU145) has been linked to high MET expression; conversely, their restoration suppresses MET expression and EMT by inhibiting the AKT/GSK-3β/Snail signaling pathway, leading to a lower metastatic potential [49,50,51,52]. Broader surveys of non-coding RNAs (ncRNAs) have described additional regulators at imprinted clusters, such as DLK1-DIO3, reinforcing the notion that the loss of various MET-targeting miRNAs collectively enhances MET signaling [53].

### 4.4. IGF-1R–MET Crosstalk

Insulin-like growth factor 1 receptor (IGF-1R) signaling can provide an alternative route for MET activation in PCa, independent of its ligand HGF. In PCa cells, IGF-1 stimulation triggered delayed MET phosphorylation through an SRC-dependent, transcriptionally mediated mechanism that requires IGF-1R activation but not HGF, suggesting a crosstalk mechanism operative in IGF1R–MET co-expressing tumors [54].

### 4.5. Autocrine and Paracrine HGF Loops

Tumor-stromal HGF overexpression can establish the formation of a paracrine loop leading to aberrant MET activation. In benign prostate tissue, HGF is predominantly produced by stromal fibroblasts; however, in prostate tumors, stromal cells and cancer-associated fibroblasts secrete increased amounts of HGF, continuously activating MET on tumor cells and driving invasive growth. ADT can further exacerbate this loop by inducing HGF production from tumor cells themselves, creating a TME that sustains MET activation, particularly in the CRPC setting. Neutralizing anti-HGF antibodies can disrupt this paracrine loop, and reduce EMT, both in vitro and in xenograft models [55].

### 4.6. Downstream Signaling and Functional Consequences

Upon HGF binding, MET activates multiple downstream pathways, including the ERK/MAPK, PI3K/AKT, SRC-family kinases and RANK/RANKL signaling pathways, which drive PCa cell proliferation, survival, motility and bone-tropic dissemination [48,56]. Transgenic MET expression in mice induced PCa tumorigenesis and cell proliferation, promoting tumor progression and metastasis [57]. In cell lines, MET signaling disrupts the E-cadherin/β-catenin complex through SRC activation and increases proteolytic cleavage of E-cadherin, in part by matrix metalloproteinases, thus reducing cell–cell adhesion and promoting EMT, evidenced by upregulation of vimentin, Snail and loss of epithelial markers [58,59,60]. Consistent with these observations, highly invasive PCa cell lines (e.g., DU145) display increased MET expression compared with less aggressive and androgen-sensitive cell lines (e.g., LNCaP). In contrast, forced overexpression of MET in LNCaP cells enhances EMT traits, tumorigenicity and bone metastasis in xenograft models via PI3K activation, whereas MET inhibition reverses these phenotypes, suggesting a causal relationship [55]. Recent investigations have further demonstrated that MET signaling cooperates with E26 transformation-specific (ETS) transcription factors to enhance ETV1/ERG-driven migration and invasion in advanced PCa models [61]. Transmembrane serine protease 2 (TMPRSS2)-ERG and TMPRSS2-ETV1 fusions, present in approximately 50% and 5–10% of PCa cases respectively, represent the most clinically relevant context for this interaction. Mechanistically, MET-activated ERK-MAPK signaling phosphorylates and stabilizes ETS factors, potentiating their transcriptional output; conversely, ERG overexpression may transcriptionally upregulate HGF/MET pathway components, potentially creating a self-amplifying loop in fusion-positive tumors. The clinical implication—that MET inhibition may be differentially effective depending on ETS fusion status—represents a hypothesis warranting prospective evaluation.

**Figure 2:**
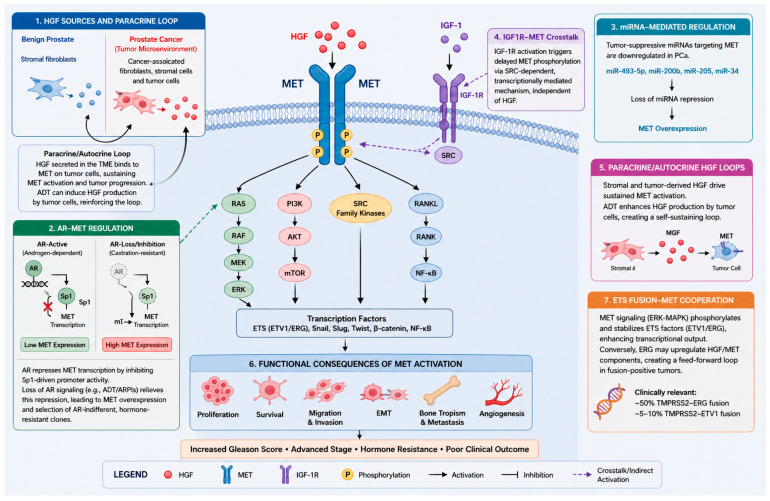
**MET signaling and regulation in prostate cancer.** Schematic overview of MET pathway regulation and downstream signaling in prostate cancer. MET activation is driven by HGF-dependent and independent mechanisms, including AR-suppression, microRNA dysregulation, IGF1R crosstalk, and paracrine/autocrine signaling within the tumor microenvironment. Activated MET engages MAPK, PI3K/AKT, SRC, and RANKL pathways, promoting proliferation, survival, migration, epithelial-to-mesenchymal transition, angiogenesis, and bone metastasis. Interactions with ETS transcription factors further enhance tumor progression.

Key preclinical models supporting the role of MET signaling in PCa progression and dissemination are summarized in Table 1, while the major regulatory networks and functional consequences of MET activation are illustrated in Figure 2.

## 5. Clinical Associations

MET activation in PCa is not uniform but enriched in distinct biological and clinical contexts. These include AR-low/AR-indifferent and NE-differentiated tumors, bone-dominant metastatic, and ARPI-resistant disease. Across these settings, MET functions as a key mediator of adaptive tumor survival, lineage plasticity, and microenvironment-driven progression [65].

MET is preferentially upregulated in AR-low and lineage-plastic subpopulations, where it supports AR-independent growth and stimulates the emergence of NE differentiation in the CRPC setting. The relationship between MET activation and lineage plasticity appears bidirectional and self-reinforcing: MET-driven EMT promotes stem-like properties (CD44-high/CD24-low phenotype) and upregulation of NE markers in experimental models, while NE-like subpopulations in turn exhibit preferential MET expression, sustaining a feed-forward loop that accelerates AR-indifferent disease progression [66]. Whether MET is sufficient to initiate full NE transdifferentiation, or whether it amplifies a program initiated by upstream events—such as retinoblastoma protein 1 (RB1)/tumor protein p53 (TP53) loss, Aurora kinase A (AURKA)/N-Myc (MYCN) activation—remains an important unresolved question. Nevertheless, combined AR and MET inhibition more effectively suppresses tumor progression and delays the expansion of resistant clones, compared with either approach alone [62], further supporting the therapeutic relevance of this axis.

Clinical evidence indicates that MET expression increases across successive stages of PCa progression and is strongly associated with bone metastasis. Immunohistochemical analyses of hormone-naïve versus hormone-refractory samples confirmed that MET expression is low to absent in many untreated primary tumors but markedly elevated in bone-metastatic disease and CRPC [45,46]. MET has been identified as one of several bypass mechanisms that sustain tumor growth despite AR inhibition, contributing to the transition from endocrine-driven to paracrine-driven signaling, promoting AR-indifferent behavior, and facilitating progression to lethal disease [65]. Consistent with these tissue-based findings, increased circulating HGF levels correlate with advanced disease stage, high tumor burden, and poor prognosis [67]. Higher preoperative serum HGF predicts lymph node metastasis and recurrence following radical prostatectomy, and both serum and urine MET levels are significantly elevated in patients with metastatic compared with localized PCa, suggesting potential utility as biomarkers beyond PSA [68,69]. The inverse correlation between MET expression and AR/PSA levels further supports a role for MET in detecting the transition to CRPC, particularly in settings where PSA fails to reflect disease burden [70]. Although these assays are not yet ready for clinical use, they underscore the translational potential of the HGF/MET axis for prognostication and disease monitoring. Collectively, these data support the HGF/MET pathway as a driver of PCa progression, metastatic dissemination, and resistance to androgen-directed therapies. Notably, MET activation is driven predominantly through transcriptional and microenvironmental rather than genomic mechanisms. In light of these observations, MET activation in PCa is best understood predominantly as an adaptive resistance mechanism. Its upregulation under therapeutic pressure supports the emergence of AR-independent disease states. However, in a subset of AR-low primary tumors with NE differentiation, MET may function as a more primary oncogenic dependency. These contextual features have direct implications for clinical development and suggest that MET may represent a therapeutically actionable target in selected disease settings.

## 6. Targeting MET in PCa

Therapeutic targeting of the HGF/MET axis in PCa has been driven by strong biological rationale [71]. Despite this, clinical translation has been disappointing, and effective targeting the HGF/MET pathway has been subject to considerable debate over the past decade. Most attempts have failed to demonstrate meaningful improvements in clinical outcomes and, in some cases, highlighted significant toxicity concerns. This limited efficacy likely reflects, at least in part, the enrollment of molecularly unselected patient populations, despite the context-dependent nature of MET activation.

### 6.1. Early Clinical Attempts: Dual AR/MET Blockade and Selective MET Inhibitors

Early attempts focused on dual AR/MET blockade, where enzalutamide was combined with crizotinib in a phase I study by Tripathi et al. PSA declines were observed in a proportion of patients, but radiographic responses were few and typically short-lived, resulting in only modest overall antitumor activity [72]. In a double-blinded phase II randomized study by Ryan et al., rilotumumab, a fully human IgG monoclonal antibody against HGF, offered no benefit when combined with mitoxantrone and prednisone (MP) versus placebo plus MP in patients with CRPC, while also suggesting a trend toward inferior OS in patients with high tumor MET expression, irrespective of treatment assignment [73]. Selective MET inhibitors have also been explored in early-phase studies. In a phase I open-label dose-escalation study by Hong et al., the oral MET inhibitor AMG 208 elicited individual complete (CR) and partial (PR) responses in heavily pretreated mCRPC, indicating early signs of biologic activity despite the small cohort and early-phase design [74]. A phase II randomized study by Monk et al. evaluated tivantinib, an orally available selective TKI that inhibits MET via a novel allosteric, ATP-independent binding mechanism, in men with asymptomatic or minimally symptomatic mCRPC. The study demonstrated manageable toxicity and significantly improved progression-free survival (PFS) compared with placebo (5.5 months vs. 3.7 months, *p* = 0.02). However, the magnitude of the benefit observed did not support further evaluation as a single agent [75].

### 6.2. Multikinase Inhibition: Sitravatinib and Cabozantinib Monotherapy

Multikinase agents such as sitravatinib have also been evaluated. In the phase 1/1b study by Bauer et al., sitravatinib yielded no objective responses but achieved disease stabilization in a subset of heavily pretreated patients, underscoring the modest efficacy of MET inhibition as monotherapy and highlighting the biological heterogeneity of pathway dependence [76,77].

Cabozantinib, an inhibitor of tyrosine kinases (TKI) targeting MET, vascular endothelial growth factor receptor 2 (VEGFR2), and other kinases, has undergone the most extensive clinical investigation. This oral agent has demonstrated the most consistent evidence of biological antitumor activity, particularly within the bone microenvironment, including high rates of bone scan improvement, pain relief, and favorable changes in circulating tumor cells (CTCs) and bone biomarkers in phase II studies [78,79,80]. Consistent with these observations, a recent phase II study in treatment-naïve patients with bone-dominant mCRPC reported substantial early disease control, with a 12-week PFS rate of 77% and bone-scan improvement in 36% of patients, reinforcing the biological relevance of MET inhibition in bone-predominant disease [81]. However, these effects did not translate into an OS benefit in the phase III COMET-1 and COMET-2 trials conducted in unselected patient populations [82,83]. This discordance between robust symptomatic and bone imaging responses, on one hand, and the lack of survival benefit, on the other, exposes a fundamental challenge in MET-directed therapy: biological activity confined to specific disease compartments, particularly the bone microenvironment, may be obscured when conventional systemic endpoints are used in molecularly unselected populations. These findings underscore major barriers to clinical development, including the absence of validated predictive biomarkers, suboptimal patient selection, and the limitations of traditional response metrics in bone-dominant disease.

### 6.3. Earlier Disease Settings

Subsequent efforts have therefore focused on earlier disease settings and rational combination strategies. In hormone-naïve metastatic disease, cabozantinib combined with ADT in a phase 2 study of 62 patients demonstrated substantial biochemical and bone responses, with a median PFS of 16.1 months. These data suggest that MET may have greater therapeutic effects earlier in the disease course, prior to the development of castration resistance [84].

### 6.4. Combination Strategies

Combination approaches in patients with mCRPC have shown greater promise. In a phase I dose escalation study, cabozantinib plus abiraterone demonstrated an acceptable safety profile and encouraging clinical activity, with a median radiographic PFS of 22 months and a median OS of 39.1 months in the 40 mg cohort, supporting further evaluation [85]. Similarly, the combination with docetaxel and prednisone in a phase I/II trial of 44 patients with mCRPC also showed manageable toxicity and improved outcomes compared with chemotherapy alone (median time to progression: 21 vs. 6.6 months; OS: 23.8 vs. 15.6 months), although the randomized component of the study was terminated early due to poor accrual [86]. More recently, integration with immune checkpoint inhibition has generated the most compelling signals. In the phase Ib COSMIC-021 study, cabozantinib (40 mg daily) combined with atezolizumab produced an overall response rate (ORR) of 23%, including 3% CR, with manageable toxicity in 132 patients previously treated with ARPIs. However, the median PFS was 5.5 months, comparable to that reported with cabozantinib monotherapy in earlier studies, leaving the incremental benefit of adding atezolizumab uncertain. These findings led to the phase 3 CONTACT-02 trial, which randomized 575 patients with mCRPC progressing after prior novel hormonal therapy to atezolizumab plus cabozantinib or second-line ARPI therapy. The study enrolled a population with poor prognostic features, including high prevalence of visceral metastasis (~48%), liver involvement (~23%), prior docetaxel exposure (~22%), and a relatively short duration of prior ARPI treatment (median ~12 months). In this adverse risk setting, the combination significantly improved PFS (6.3 vs. 4.2 months; HR 0.65; *p* = 0.0007) and ORR compared with ARPI switching, although no survival advantage has been reported to date [87,88].

Taken together, the available evidence suggests that the clinical activity of MET inhibition lies not in broad application but in biologically selected disease contexts, particularly when MET is incorporated into combination strategies targeting complementary tumor-intrinsic and microenvironmental pathways. The consistent failure of MET inhibitors in unselected populations does not invalidate MET as a target; rather, it highlights a fundamental mismatch between drug mechanism and trial design. A comprehensive summary of MET-targeted clinical studies and their key outcomes is provided in Table 2.

## 7. Mechanisms of Resistance to MET Pathway Inhibition in PCa

Resistance to MET pathway inhibition in PCa arises through a combination of tumor-intrinsic adaptations and microenvironment-driven mechanisms. Although MET blockade can induce initial tumor regression, MET signaling within tumor cells is not a durable therapeutic dependency, as clusters of viable phosphorylated MET (pMET)-positive cells often persist and rapidly regrow under selective pressure. The antitumor activity of MET/VEGFR2 inhibitors is largely mediated through the suppression of angiogenesis and the modulation of the osteoblastic niche, rather than through the sustained inhibition of MET in cancer cells. Vascular heterogeneity, particularly the presence of VEGFR2-negative vessels capable of maintaining residual tumor foci, represents a key driver of primary resistance to MET-targeted therapy [64]. In parallel, chronic MET inhibition promotes bypass signaling, most prominently through fibroblast growth factor receptor 1 (FGFR1) upregulation. This process, transcriptionally regulated by Yes-associated protein (YAP) and T-box transcription factor 5 (TBX5), enables tumor cells to sustain proliferation and survival via alternative receptor tyrosine kinase pathways [63]. Collectively, these findings indicate that resistance to MET inhibition reflects both microenvironmental protection and dynamic signaling rewiring, highlighting the need for combinatorial or multitargeted therapeutic strategies. Key preclinical models of resistance to MET inhibition are also summarized in Table 1.

## 8. Current Limitations and Challenges

**Figure 3:**
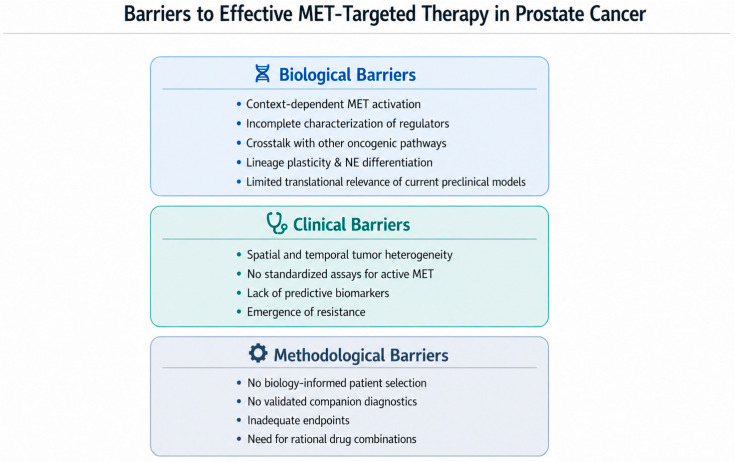
**From biological complexity to clinical impact: barriers to effective clinical drug development.** Conceptual framework illustrating the biological complexity, clinical heterogeneity, and methodological limitations that contribute to the disconnect between strong biological rationale and modest clinical efficacy.

Targeting the MET pathway represents a compelling but complex opportunity in the management of PCa, and its future success will depend on an integrative approach combining molecular diagnostics, rational drug combinations, and precision clinical trial design. However, significant knowledge gaps and challenges inherent to the complexity of MET biology have impeded the translation of promising preclinical findings into consistent clinical benefits for patients with PCa (Figure 3).

### 8.1. Biological Challenges

Context-dependent MET activation is among the most critical barriers, rendering pathway dependence variable across disease states and patient subgroups. The intricate crosstalk between MET signaling and other oncogenic pathways—including AR, PI3K/AKT, and immune-related networks—further complicates therapeutic targeting, as MET inhibition alone may be insufficient to suppress a broader and interconnected signaling network. The incomplete characterization of upstream regulators and downstream effectors of MET activation limits our understanding of its precise role in tumor progression, therapeutic resistance, and metastatic dissemination. Current preclinical PCa models represent an additional limitation, as they often fail to recapitulate the full biological and molecular heterogeneity of human disease, reducing the translational relevance of preclinical findings. Within this framework, specific molecular interactions remain insufficiently defined. In particular, the interplay between AR splice variants—most notably androgen receptor splice variant 7 (AR-V7)—and MET transcriptional regulation remains unclear. As AR-V7 lacks the ligand-binding domain required for AR-mediated repression of MET, its impact on MET expression and pathway dependency in the ARPI-resistant setting warrants further investigation. Finally, the molecular mechanisms by which MET contributes to lineage plasticity and the emergence of AR-independent or NE phenotypes require further clarification—most critically, whether MET functions as a driver or an amplifier of NE transdifferentiation. Resolving this question might reveal novel and therapeutically exploitable vulnerabilities.

### 8.2. Clinical Challenges

At the clinical level, spatial and temporal tumor heterogeneity poses a fundamental challenge: MET expression and activity vary across metastatic sites and evolve dynamically under therapeutic pressure, making point-in-time assessments potentially unrepresentative of the broader disease state. Standardized, clinically validated assays to assess biologically relevant MET activity in individual patients are currently lacking. This impedes the development of robust predictive biomarkers and constrains the ability to identify patients most likely to benefit from MET-targeted therapies. The emergence of resistance further limits the durability of single-agent MET inhibition and underscores the need for rational combination approaches.

### 8.3. Methodological Challenges

A key limitation of prior MET-targeted trials has been the use of conventional systemic endpoints that proved insufficient to capture the compartment-specific activity of MET inhibition, particularly within the bone microenvironment; PSA response and PFS alone may underestimate the full therapeutic benefit. The absence of biology-informed patient selection and validated companion diagnostics led to the enrollment of molecularly unselected populations, diluting therapeutic signals and obscuring clinically relevant responder subgroups.

## 9. Future Perspectives on Targeting the MET Pathway in PCa

### 9.1. Biomarker Development and Patient Selection

The available evidence indicates that the clinical development of MET inhibition in PCa should shift from a broad, unselected approach toward precision strategies enriching for biological contexts in which MET signaling is most relevant. Emerging data suggest that MET upregulation is particularly important in AR-indifferent phenotypes, treatment-emergent NE-PCa, bone-dominant metastatic disease, early castration resistance following ARPI therapy, and in tumors with hypoxic or immunosuppressive microenvironments, where traditional hormone-targeted strategies fail. This concept is supported by clinical observations from several MET-directed trials in mCRPC, in which patients with aggressive, high volume metastatic disease demonstrated the most pronounced therapeutic signals. These results indicate that MET may be a context-specific therapeutic driver that is highly active in certain biologically enriched subgroups but not in unselected populations [76,77].

From a translational perspective, a critical priority is the development of robust biomarkers capable of identifying MET-dependent tumors. Tissue-based approaches remain the most established, including MET and pMET immunohistochemistry, as well as transcriptomic signatures associated with AR-indifferent disease states. These strategies provide direct insight into pathway expression and activation within tumor tissue, potentially reflecting MET pathway dependence.

Liquid biopsy approaches provide complementary, minimally invasive tools for monitoring disease biology. Circulating HGF levels correlate with prognosis and CTC positivity with CRPC transition [67,80]. Circulating tumor DNA (ctDNA) profiling may detect MET pathway alterations longitudinally. Exosomal HGF/MET profiling represents an emerging biofluid-based monitoring strategy. Together, these approaches enable adaptive treatment strategies and prospective tracking of MET pathway activation.

Finally, molecular imaging techniques, particularly MET-specific PET tracers, represent an emerging modality that may allow real-time, whole-body assessment of MET expression and activity across disease sites. This approach may support both patient selection and treatment monitoring, with particular relevance in bone-dominant disease.

### 9.2. Emerging Therapeutic Strategies

Growing interest in dual- or multitarget approaches reflects the recognition that MET inhibition alone is unlikely to achieve durable responses in the face of dynamic signaling rewiring. Combining MET inhibition with other agents could overcome compensatory mechanisms of resistance and enhance therapeutic efficacy in selected patient subsets. Preclinical studies have demonstrated synergistic effects when MET inhibitors are combined with PARP inhibitors in tumors exhibiting co-alterations in MET and DNA repair genes, and with antiangiogenic compounds in tumors with hypoxic and immune-suppressive microenvironments [89,90]. Building on the established resistance mechanisms, co-targeting FGFR1 may be required to prevent emergent bypass signaling, particularly in bone-dominant disease [63]. In the context of NE differentiation and emergence of lineage plasticity, MET inhibition combined with Aurora kinase or EZH2 inhibitors may prove synergistic by simultaneously targeting MET-driven survival signaling and the epigenetic reprogramming that sustains AR-indifferent disease states.

Beyond conventional TKI combinations, the concept of MET-directed payload delivery represents an unexplored but mechanistically attractive strategy in PCa. By exploiting MET surface expression to deliver cytotoxic or radionuclide payloads directly to MET-high tumor cells, this approach bypasses the downstream signaling rewiring that limits sustained kinase inhibition. Telisotumab Vedotin, a MET-directed antibody-drug conjugate currently in clinical development for MET-overexpressing solid tumors, has not yet been evaluated in PCa. Similarly, MET-targeted radioligand therapy—conceptually analogous to ^177^Lu-PSMA-617—remains unexplored in PCa, despite MET enrichment in bone metastases, making it a particularly compelling candidate. Both strategies represent rational hypotheses warranting prospective investigation.

### 9.3. Trial Design Priorities

**Figure 4:**
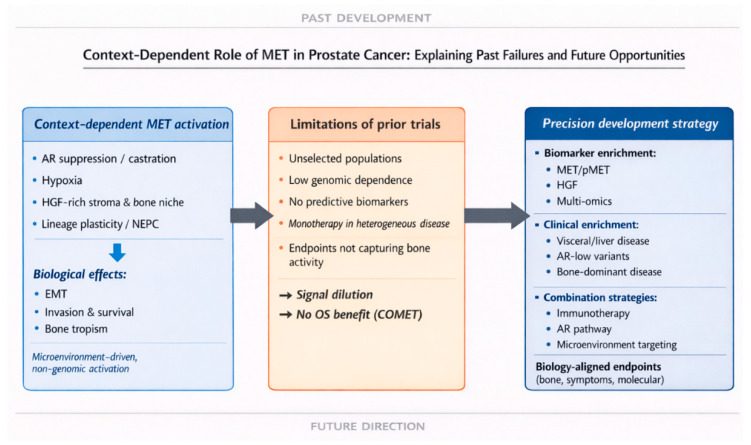
**Context-dependent MET signaling and translational implications in prostate cancer.** MET activation in prostate cancer is largely microenvironment-driven and non-genomic, promoting invasion, survival, and bone tropism. Prior trials in unselected populations using conventional endpoints led to signal dilution and limited survival benefit. Future development should prioritize biomarker-enriched patient selection, clinical enrichment for aggressive disease, rational combinations, and biology-aligned endpoints.

As precision oncology evolves, integrating MET-targeted strategies into biomarker-driven care holds promise for improving outcomes in advanced PCa. However, successful translation into clinical practice will require carefully designed, biomarker-enriched clinical trials in which bone-focused endpoints are routinely incorporated to validate efficacy and identify resistance mechanisms. Specifically, future research should aim to (1) define and validate context-dependent MET-driven molecular subtypes using functional genomics; (2) apply multi-omics approaches to develop blood-based and tissue-based biomarker assays for monitoring MET pathway activation both within the TME and the systemic level; (3) use these tools to stratify patients according to their MET activation status and to identify those in whom MET drives disease progression; and (4) design and conduct biology-driven, biomarker-enriched proof-of-concept clinical trials that incorporate clinical enrichment for MET-relevant disease contexts—such as visceral or liver metastases, bone-dominant disease, early resistance to ARPIs, and AR-low phenotypes—together with endpoints tailored to MET biology, including bone-specific, symptom-based, and molecular response metrics (Figure 4).

## 10. Conclusions

As resistance to ADT and next-generation ARPIs becomes increasingly prevalent, alternative oncogenic pathways will play a greater role in disease evolution. Preclinical studies indicate that the MET signaling pathway contributes to PCa progression and metastatic dissemination, particularly in aggressive disease states. Clinically, MET overexpression and activation are associated with adverse pathological features, poor prognosis, enhanced EMT, and increased metastatic potential.

Despite these compelling observations, the clinical efficacy of MET-targeted therapies in PCa has been modest, underscoring the complexity and context-dependent nature of MET pathway activation and therapeutic relevance. Moving forward, a more comprehensive molecular characterization of MET dysregulation across PCa subtypes is essential. This includes elucidating the interplay between MET and AR signaling, as well as its role in lineage plasticity and microenvironmental crosstalk. Identifying the clinical and molecular contexts associated with MET pathway dependence and developing predictive biomarkers to stratify patients will be critical for effective patient selection. The future clinical impact of MET-directed therapy will depend on integrating biomarker-enriched strategies with rational combination approaches targeting complementary tumor-intrinsic and microenvironmental pathways.

In this context, MET should be regarded as a context-dependent vulnerability rather than a universal therapeutic target.

## Figures and Tables

**Table 1:** Preclinical models supporting the role of MET signaling in prostate cancer progression, epithelial-to-mesenchymal transition, metastasis, and therapeutic resistance. The table summarizes key experimental systems, MET-related mechanisms, and major functional outcomes.

Model System	Key Intervention	MET-Related Mechanism/Context	Major Finding	References
LNCaP (AR+) cells/xenograft	MET overexpression	Forced MET overexpression	Promotes epithelial-to-mesenchymal transition (EMT), tumorigenicity, and bone metastasis via PI3K activation; effects reversed by MET inhibition	Han c., 2014 [55]
DU145 (AR−) cells	MET knockdown/inhibition	Baseline MET-high expression state	MET inhibition reduces invasion and EMT in MET-high cells	Putzke et al., 2011 [58]
PC3, DU145 (AR−) cells	miRNA restoration (miR-493-5p, miR-200b, miR-205, miR-34c)	MET-high/miRNA-low expression state	Restoration suppresses MET and EMT via AKT/GSK-3β/Snail signaling; reduces metastatic potential	Hagman et al., 2013; Wang et al., 2017; Williams et al., 2013; Chauhan et al., 2022 [49,50,51,52]
Transgenic murine model	Prostate-specific MET overexpression	MET overactivation	Induces tumorigenesis, promotes progression and metastasis	Mi et al., 2018 [57]
LNCaP/castration-resistant prostate cancer (CRPC) cell lines	Cabozantinib ± enzalutamide	MET upregulation following AR suppression	Combined MET and AR inhibition more effectively suppresses tumor growth than either agent alone; delays emergence of resistant clones	Qiao et al., 2016 [62]
LNCaP, LREX, 905L, CRPC cell lines/xenograft	Cabozantinib	Adaptive resistance via FGFR1 bypass	YAP/TBX5-driven FGFR1 upregulation mediates resistance; VEGFR2-negative vessels sustain residual tumor cell survival	Koinis et al., 2020 [63]
Murine bone metastasis model	Cabozantinib	MET/VEGFR2 inhibition	Suppression of angiogenesis and osteoblastic niche; VEGFR2-negative vessels drive primary resistance	Varkaris et al., 2016 [64]

Abbreviations: EMT: epithelial-to-mesenchymal transition; AR: androgen receptor; CRPC: castration-resistant prostate, micro-RNA: miRNA, FGFR1: fibroblast growth factor receptor 1, VEGFR2: vascular endothelial growth factor receptor 2, YAP: Yes-associated protein, TBX5: T-box transcription factor 5.

**Table 2:** Summary of selected ongoing and completed clinical trials evaluating MET-targeted therapies in prostate cancer. Key study characteristics and efficacy outcomes are presented.

Reference	Country	Trial Phase	Title of the Trial	Intervention	Study Population	Response Rates	Study Status	Clinical Trial Identifier
Tripathi A. et al., Clinical Cancer Research, 2020 [72]	USA (1 location)	Phase I, non- randomized, Interventional	Crizotinib in Combination with Enzalutamide in Metastatic Castration-resistant Prostate Cancer	Fixed-dose enzalutamide 160 mg QD combined with crizotinib at three dose levels: Dose Level 1: 250 mg QD, Dose Level 2: 200 mg BID, Dose Level 3: 250 mg BID	-24 patients with metastatic castration-resistant prostate cancer (mCRPC) -ECOG performance status < 2 -Radiographic evidence of metastatic disease -Evidence of disease progression based on rising PSA levels -No limit on the number of prior lines of therapy	-PSA declines ≥ 50% observed in 33–38% of patients (≥90% in 25–27%), though not confirmed or durable -Best radiographic response: PR 12–13%, SD 40–46% -Median PFS: 5.5 months	Completed	NCT02207504
Monk P. et al., Invest New Drugs, 2018 [75]	USA (20 locations)	Phase II, randomized, Interventional	Tivantinib in Treating Patients with Metastatic Prostate Cancer	Tivantinib 360 mg BID vs. placebo, randomized 2:1	-Patients: 80 men with asymptomatic or minimally symptomatic mCRPC -ECOG performance status 0–1 -Radiographic evidence of metastatic disease (bone ± soft tissue) -Prior therapy: at least one androgen deprivation regimen, no prior chemotherapy for mCRPC	-Median PFS: 5.5 months (tivantinib) vs. 3.7 months (placebo), HR 0.55 (*p* = 0.02) -PSA response (≥50% decline): 7 patients (12%) vs. 3 patients (11%) -Objective radiographic response (RECIST 1.1): 4 patients (7%) vs. 2 patients (7%) -Disease control rate (SD + PR): 56% vs. 44%	Completed	NCT01519414
Bauer T et al., Invest New Drugs, 2022 [76,77]	USA, South Korea, (International, 43 locations)	Phase 1/1b, non-randomized, Interventional	Phase 1/1b Study of MGCD516 in Patients with Advanced Cancer	Sitravatinib (MGCD516) 120 mg QD, oral, continuous 28-day cycles, administered until disease progression or unacceptable toxicity	-Cohorts: Advanced solid tumors (Phase 1a) and disease-specific expansions (Phase 1b) *-n* = 193 -Median age: 67 years -ECOG performance status: 0–1 -Median prior systemic regimens: 3 (range 1–6) -All had prior androgen-receptor pathway inhibition and/or chemotherapy	-Objective response rate (ORR): 0% (no confirmed partial or complete responses) -Stable disease (SD): 44% of CRPC patients achieved SD as best response -Median progression-free survival (PFS): 5.8 months -Median overall survival (OS): 10.1 months	Completed	NCT02219711
Smith M et al., Journal of Clinical Oncology, 2016 [82]	USA, Europe, Australia (International, 264 locations)	A Phase 3, Randomized, Double-blind, Controlled Study, Interventional	Study of Cabozantinib (XL184) Versus Prednisone in Men with Metastatic Castration-resistant Prostate Cancer Previously Treated with Docetaxel and Abiraterone or MDV3100 (COMET-1)	Cabozantinib (XL184) 60 mg QD vs. Prednisone 5 mg BID, randomized 2:1, oral, continuous dosing until progression or toxicity	*-n* = 1.028 (682 cabozantinib, 346 prednisone) men with mCRPC -Progressive disease after docetaxel + abiraterone and/or enzalutamide -ECOG 0–2, median age 69 years -100% with bone metastases, 20% with visceral disease	-Bone scan response (BSR 12 weeks): 42% vs. 3% -Median rPFS: 5.6 vs. 2.8 months (HR 0.48) -Median OS: 11.0 vs. 9.8 months (HR 0.90) -PSA response ≥ 50%: 6% vs. 2% -circulating tumor cells conversion: 33% vs. 6%	Completed	NCT01605227
Corn PG et al., Clinical Cancer Research, 2020 [84]	USA (1 location)	Phase II, Open-label, Single-arm, Interventional	Cabozantinib and Androgen Ablation in Patients with Androgen-Dependent Metastatic Prostate Cancer	Cabozantinib 60 mg QD combined with androgen deprivation therapy (ADT) in treatment-naïve metastatic prostate cancer (hormone-naïve). Continuous 28-day cycles until progression or toxicity	*-n* = 36 men with hormone-naïve metastatic prostate cancer (mHNPC) -ECOG 0–1, median age 65 years -Radiographic evidence of metastatic disease -No prior systemic therapy for metastatic disease (≤3 months ADT allowed) -Concurrent ADT (leuprolide or degarelix) in all patients	-PSA90 decline: 83% of evaluable patients -PSA50 decline: 94% of evaluable patients -Radiographic response: 90% (1 CR, 8 PR among 10 evaluable) -Bone scan improvement: 81% -Median time to CRPC: 16.1 months	Completed	NCT01630590
Ryan CJ et al., Clinical Cancer Research, 2013 [73]	USA, Europe, Australia (international) *	Phase II, Double-blinded study Randomized, Interventional	AMG 102 in Combination with Mitoxantrone and Prednisone in Subjects with Previously Treated Castrate Resistant Prostate Cancer	Rilotumumab (AMG 102), a fully human monoclonal antibody against HGF, 7.5 mg/kg or 15 mg/kg IV every 3 weeks, combined with Mitoxantrone 12 mg/m^2^ IV day 1 and Prednisone 5 mg BID Randomized 1:1:1 (Rilotumumab 7.5 mg/kg + MP vs. 15 mg/kg + MP vs. placebo + MP), up to 12 cycles or until progression/toxicity	*-n* = 144 patients with taxane-refractory metastatic castration-resistant prostate cancer (mCRPC) -ECOG 0–1, median age 67 years (range 48–87) -Radiographic evidence of metastatic disease -Progressive disease by PSA, RECIST 1.0, or new bone lesions -Prior taxane-based chemotherapy required, ≤1 prior regimen for CRPC	-Median OS: 12.2 months (vs. 11.1 months control), HR 1.10 (80% CI 0.82–1.48) -Median PFS: 3.0 months (vs. 2.9 months control), HR 1.02 (80% CI 0.79–1.31) -PSA response (≥50% decline): 11% (vs. 14% control) -Objective response rate: 0%, Stable disease: 37% (vs. 43%) -No bone scan responses reported	Completed	NCT00770848
Hong DS et al., Oncotarget, 2015 [74]	USA (3 locations)	Phase I, open-label, sequential dose escalation, Interventional	A Phase 1 Study of AMG 208 in Subjects with Advanced Solid Tumors	AMG 208 (oral MET inhibitor) dose-escalation 5 mg–40 mg QD until progression or unacceptable toxicity	-11 patients with mCRPC -ECOG 0–2, median age 63 years -All had progressive disease after standard therapy	In the CRPC subset: -1 complete response (CR) -2 partial responses (PR) -4 patients with stable disease (SD)	Completed	NCT00813384
Smith MR et al., Journal of Clinical Oncology, 2014 [78,79,80]	USA, Europe, Asia (multi-center, 47 locations)	Phase 2, Randomized, Interventional	Study of Cabozantinib (XL184) in Adults with Advanced Malignancies	Cabozantinib 100 mg QD during 12-week open-label lead-in patients with stable disease randomized to cabozantinib vs. placebo (Randomized Discontinuation Trial)	-171 men with mCRPC -measurable disease (RECIST 1.0) -ECOG 0–1 -prior chemotherapy required -radiographic progression at baseline -exclusions: PSA-only progression, brain metastases	-Soft-tissue regression: 72%, ORR 5%, SD 75% -Bone scan improvement: 68% (12% CR) -Pain improvement: 67%, reduced narcotic use: 56% -Median PFS after randomization: 23.9 vs. 5.9 weeks (HR 0.12, *p* < 0.001)	Completed	NCT00940225
Agarwal N et al., Lancet Oncology, 2025 [87]	USA, Europe, Asia- Pacific, Latin America, Australia (280 locations)	Phase 3, randomized, open-label, controlled study, Interventional	Study of Cabozantinib in Combination with Atezolizumab Versus Second NHT in Subjects With mCRPC (CONTACT-02)	Cabozantinib 40 mg QD + atezolizumab 1200 mg IV every 3 weeks vs. ARPI switch (abiraterone 1000 mg QD + prednisone 5 mg BID, or enzalutamide 160 mg QD)	*-n* = 575 men with mCRPC -Measurable extrapelvic soft-tissue metastases (lymph-node or visceral) per RECIST 1.1 -Progression on one prior ARPI (mostly in the mCRPC setting) -ECOG performance status 0–1, median age 71 years -Visceral metastases in 48%, liver metastases in 23%, bone metastases in 79% -Prior docetaxel for mHSPC allowed (~22% of patients)	-Median PFS: 6.3 vs. 4.2 months (HR 0.65, 95% CI 0.50–0.84; *p* = 0.0007) -Median OS: 14.8 vs. 15.0 months (HR 0.89, 95% CI 0.72–1.10; *p* = 0.30) -ORR: 13% vs. 6% -Disease control rate: 72% vs. 53% -PSA response ≥ 50%: 14% vs. 15%	Active, not recruiting	NCT04446117
Agarwal N et al., Lancet Oncology, 2022 [88]	USA, Europe, Australia (International, 124 locations)	Phase 1/1b, non- randomized, open label, Interventional	Study of Cabozantinib Alone or in Combination with Atezolizumab to Subjects with Locally Advanced or Metastatic Solid Tumors (COSMIC-021)	Cabozantinib 40 mg QD (oral) + Atezolizumab 1200 mg IV every 3 weeks, continuous dosing until radiographic/clinical progression or unacceptable toxicity (Dose-escalation stage allowed 40–60 mg cabozantinib; expansion cohort used 40 mg)	*-n* = 132 men with mCRPC -Radiographic soft tissue progression on/after enzalutamide or abiraterone (or both) -Measurable soft tissue disease (RECIST 1.1 requirement) -ECOG 0–1 -Chemotherapy for mCRPC not allowed (prior docetaxel in hormone-sensitive setting permitted)	-Objective response rate (ORR): 23% (3% CR, 21% PR) -Disease control rate: 84% -Median duration of response: 8.3 months -Median progression-free survival (PFS): 5.5 months (95% CI 4.3–6.6) -Median overall survival (OS): 18.4 months -PSA decline ≥ 50%: 23% of evaluable patients	Active, not recruiting	NCT03170960
Madan RA et al., BJU Int, 2022 [86]	USA (1 location)	Phase 1/2, Randomized, open-label, Interventional	Cabozantinib Plus Docetaxel and Prednisone for Advanced Prostate Cancer	Phase 1: Cabozantinib 20–40 mg QD (dose-escalation) + Docetaxel 75 mg/m^2^ IV + Prednisone 5 mg BID, continuous 28-day cycles. Phase 2: Cabozantinib 40 mg QD + Docetaxel 75 mg/m^2^ IV q3w + Prednisone 5 mg BID vs. Docetaxel/Prednisone alone, until disease progression or unacceptable toxicity	-Phase 1: 19 patients -Phase 2: 13 combination vs. 12 control -Median age: phase 1: 67 years, phase 2: 69 years -ECOG 0–2 (majority ECOG 1) -All with radiographic evidence of metastatic CRPC -Prior therapies: abiraterone and/or enzalutamide, chemotherapy (≤2 patients)	-Phase 1: Median TTP 13.6 months, Median OS 16.3 months -Phase 2: Median TTP 21.0 vs. 6.6 months (*p* = 0.035), Median OS 23.8 vs. 15.6 months (*p* = 0.072) favoring combination	Completed	NCT01683994
Smith DC et al., Clin. Genitourin Cancer, 2020 [81]	USA (1 location)	Phase 2, non- randomized, Interventional	Trial of Cabozantinib (XL184) in Castrate-Resistant Prostate Cancer Metastatic to Bone	Cabozantinib 60 mg QD, oral, continuous 28-day cycles until progression or intolerable toxicity	*-n* = 22 evaluable men with treatment-naïve mCRPC (no prior docetaxel, abiraterone, or enzalutamide) -ECOG 0–1 -median age ~ 68 years -All had bone metastases	-12-week PFS rate: 77% -Median PFS: 43.7 weeks (95% CI 23.7–97.0) -Bone-scan improvement: 8/22 (36%) -PSA response ≥ 50%: 4 patients (18%) -Median time on treatment: 24 weeks	Terminated	NCT01428219
Basch EM et al., European Urology Supplements, 2019 [83]	USA, Europe, Australia (International, 82 locations)	Phase 3, Randomized, Double-blind, Controlled Trial, Interventional	Study of Cabozantinib (XL184) Versus Mitoxantrone Plus Prednisone in Men with Previously Treated Symptomatic Castration-resistant Prostate Cancer (COMET-2)	Cabozantinib 60 mg QD (oral) vs. Mitoxantrone 12 mg/m^2^ IV q3w + Prednisone 5 mg BID, randomized 1:1	*-n* = 119 patients with mCRPC presenting with clinically significant bone pain following prior therapies (docetaxel and abiraterone or enzalutamide) -ECOG 0–2, median age ~ 68 years -Radiographic bone metastases in all patients -Exclusion: visceral crisis or impending spinal-cord compression	-pain response: 15% (cabozantinib) vs. 17% (control) -Median PFS: 5.6 months (cabozantinib) vs. 2.8 months (control) (HR 0.50, *p* < 0.001) -Median OS: 9.0 months vs. 11.0 months (HR 1.05, *p* = 0.81) -Bone-scan improvement: 42% vs. 3%	Terminated	NCT01522443

Abbreviations: mCRPC: metastatic castration-resistant prostate cancer, mHNPC: metastatic hormone-naïve prostate cancer, mHSPC: metastatic hormone-sensitive prostate cancer, CRPC: castration-resistant prostate cancer, ADT: androgen deprivation therapy, ARPI: androgen receptor pathway inhibitor, PD: progressive disease, SD: stable disease, PR: partial response, CR: complete response, ORR: objective response rate, DCR: disease control rate, PFS: progression-free survival, OS: overall survival, TTP: time to progression, QD: once daily, BID: twice daily, IV: intravenous, q3w: every 3 weeks, RECIST 1.1: Response Evaluation Criteria in Solid Tumors version 1.1, BSR: bone scan response, PSA: prostate-specific antigen, NHT: novel hormonal therapy, HR: hazard ratio, CI: confidence interval, Mets: metastases. * Reported as multi-center and listed as international in ClinicalTrials.gov; however, participating countries and number of study sites were not specified in the publication or registry entry.

## Data Availability

The data presented in this study are available upon request from the corresponding author.

## Abbreviations

The following abbreviations are used in this manuscript:

PCa	Prostate cancer
OS	Overall survival
AR	Androgen receptor
mCRPC	Metastatic castration-resistant prostate cancer
PSMA	Prostate-specific membrane antigen
PARP	Poly(ADP-ribose) polymerase
HGF	Hepatocyte growth factor
NE	Neuroendocrine
TKI	Inhibitors of tyrosine kinases
PI3K	Phosphoinositide 3-kinase
PLCγ1	Phospholipase Cγ1
GRB2	Growth factor receptor-bound protein 2
GAB1	GRB2-associated binding protein 1
STAT3	Signal transducer and activator of transcription 3
SHC	Src homology-2-containing
CRK	V-crk sarcoma virus CT10 oncogene homolog
CRKL	CRK-like
SRC	V-src sarcoma viral oncogene homolog
SHIP-2	Src homology domain-containing 5′ inositol phosphatase
MAPK	Mitogen-activated protein kinase
FAK	Focal adhesion kinase
CBL	Casitas B lineage lymphoma
EGFR	Epidermal growth factor receptor
TME	Tumor microenvironment
HIF-1α	Hypoxia inducible factor 1-alpha
EMT	Epithelial-to-mesenchymal transition
PIN	Prostatic intraepithelial neoplasia
ADT	Androgen deprivation therapy
ARPIs	AR pathway inhibitors
miRNAs	MicroRNAs
ncRNAs	Non-coding RNAs
IGF-1R	Insulin-like growth factor 1 receptor
ETS	E26 transformation-specific
TMPRSS2	Transmembrane Protease Serine 2
RB1	Retinoblastoma protein 1
TP53	Tumor protein p53
AURKA	Aurora kinase A
MYCN	N-Myc
MP	Mitoxantrone and prednisone
CR	Complete response
PR	Partial response
ORR	Overall response rate
PFS	Progression-free survival
CTCs	Circulating tumor cells
AR-V7	Androgen receptor splice variant 7
ctDNA	Circulating tumor DNA
pMET	Phosphorylated-MET
FGFR1	Fibroblast growth factor receptor 1
YAP	Yes-associated protein
TBX5	T-box transcription factor 5
